# Retraction notice for: Characteristics of liver fibrosis with different etiologies using a fully quantitative fibrosis assessment tool [Braz J Med Biol Res (2017) 50(6): e5234]

**DOI:** 10.1590/1414-431X20175234retraction

**Published:** 2017-07-03

**Retraction for:** Braz J Med Biol Res | doi: 10.1590/1414-431x20175234 | PMID: 28538834

The authors would like to retract the article “Characteristics of liver fibrosis with different etiologies using a fully quantitative fibrosis assessment tool” that was published in volume 50 no. 6 (2017) (Epub May 18, 2017) in the *Brazilian Journal of Medical and Biological Research* <http://dx.doi.org/10.1590/1414-431x20175234> PMID: 28538834.

